# Real-world performance analysis of a novel computational method in the precision oncology of pediatric tumors

**DOI:** 10.1007/s12519-023-00700-2

**Published:** 2023-03-13

**Authors:** Barbara Vodicska, Júlia Déri, Dóra Tihanyi, Edit Várkondi, Enikő Kispéter, Róbert Dóczi, Dóra Lakatos, Anna Dirner, Mátyás Vidermann, Péter Filotás, Réka Szalkai-Dénes, István Szegedi, Katalin Bartyik, Krisztina Míta Gábor, Réka Simon, Péter Hauser, György Péter, Csongor Kiss, Miklós Garami, István Peták

**Affiliations:** 1 Oncompass Medicine Hungary Kft, Retek Str. 34, Budapest, 1024 Hungary; 2 Division of Pediatric Hematology-Oncology, Department of Pediatrics, Faculty of Medicine, University of Debrecen, Debrecen, Hungary; 3 Department of Pediatrics, Albert Szent-Györgyi Medical School, University of Szeged, Szeged, Hungary; 4 Onco-Hematology Department, Velkey László Paediatric Health Centre, Miskolc, Hungary; 5 Department of Paediatrics, Semmelweis University, Budapest, Hungary; 6 Onco-Hematology Department, Heim Pál Children’s Hospital, Budapest, Hungary; 7 Department of Pharmacology and Pharmacotherapy, Semmelweis University, Budapest, Hungary; 8 Department of Pharmaceutical Sciences, University of Illinois at Chicago, Chicago, USA; 9 Genomate Health, Cambridge, MA USA

**Keywords:** Computational decision support, Pediatric tumors, Precision oncology, Tumor board

## Abstract

**Background:**

The utility of routine extensive molecular profiling of pediatric tumors is a matter of debate due to the high number of genetic alterations of unknown significance or low evidence and the lack of standardized and personalized decision support methods. Digital drug assignment (DDA) is a novel computational method to prioritize treatment options by aggregating numerous evidence-based associations between multiple drivers, targets, and targeted agents. DDA has been validated to improve personalized treatment decisions based on the outcome data of adult patients treated in the SHIVA01 clinical trial. The aim of this study was to evaluate the utility of DDA in pediatric oncology.

**Methods:**

Between 2017 and 2020, 103 high-risk pediatric cancer patients (< 21 years) were involved in our precision oncology program, and samples from 100 patients were eligible for further analysis. Tissue or blood samples were analyzed by whole-exome (WES) or targeted panel sequencing and other molecular diagnostic modalities and processed by a software system using the DDA algorithm for therapeutic decision support. Finally, a molecular tumor board (MTB) evaluated the results to provide therapy recommendations.

**Results:**

Of the 100 cases with comprehensive molecular diagnostic data, 88 yielded WES and 12 panel sequencing results. DDA identified matching off-label targeted treatment options (actionability) in 72/100 cases (72%), while 57/100 (57%) showed potential drug resistance. Actionability reached 88% (29/33) by 2020 due to the continuous updates of the evidence database. MTB approved the clinical use of a DDA-top-listed treatment in 56 of 72 actionable cases (78%). The approved therapies had significantly higher aggregated evidence levels (AELs) than dismissed therapies. Filtering of WES results for targeted panels missed important mutations affecting therapy selection.

**Conclusions:**

DDA is a promising approach to overcome challenges associated with the interpretation of extensive molecular profiling in the routine care of high-risk pediatric cancers. Knowledgebase updates enable automatic interpretation of a continuously expanding gene set, a “virtual” panel, filtered out from genome-wide analysis to always maximize the performance of precision treatment planning.

**Supplementary Information:**

The online version contains supplementary material available at 10.1007/s12519-023-00700-2.

## Introduction

Genome-scale next-generation sequencing (NGS) methods such as whole-exome or whole-genome sequencing (WES/WGS) have provided significant insight into the molecular background of cancer [1]. Due to the potential for improved outcomes, an increasing number of oncologists have adopted a precision medicine approach based on the notion that treatment with matched targeted therapy can have superior clinical activity [2–6].

In contrast, the possibilities for implementing personalized treatment in pediatric cancer care are rather limited, although cancer is the primary cause of death by disease in children past infancy [7]. There is a high response rate to chemotherapies, and only a few targeted drugs are approved for children. However, high-risk, relapsed or refractory pediatric cancers have poor prognoses and cannot be effectively treated, as indicated by the 20% five-year overall survival rate for children with relapsed neuroblastoma [8, 9]. Therefore, a number of institutions have started paving the way for pediatric precision oncology by determining the value of comprehensive molecular profiling for children’s tumors [10, 11]. Such initiatives have detected actionable findings in 15%–87% of cases [12–35] (Table 1). Moreover, it has been shown that targeted therapy might improve outcomes in a portion of pediatric patients [19, 20, 36–40].

**Table 1:** Published pediatric sequencing studies with cases > 50 including actionable finding ratios

Studies	Sequencing methods	Actionability criteria	Actionability^a^	Patients	Year	References
BASIC3	Tumor + normal WES	Somatic mutations of (potential) clinical utility, mutations in consensus cancer genes	47% (*n* = 121)	Children, newly diagnosed solid tumors	12–14	[28]
PIPseq	Tumor + normal WES, RNAseq	Approved or experimental drug inhibiting the target or its downstream pathway; or preclinical evidence with age-appropriate dosing information	38% (*n* = 101)	Children, high-risk cancer, and hematologic disorders	14–16	[12]
PIPseq Hem	Tumor + normal WES or NGS (467 genes), RNAseq	Approved or experimental drug inhibiting the target or its downstream pathway; or preclinical evidence with age-appropriate dosing information	80% (*n* = 56)	Children, hematologic malignancies, blood disorders	14–17	[17]
SMPAEDS	Tumor + normal NGS (80–90 genes)	OncoKB, COSMIC mutations/SNVs, CNVs, for which a clinical trial was recruiting; biomarker predictive of drug response	51% (*n* = 209)	≤ 24 y, solid tumors	16–18	[13]
MBB Program	Tumor NGS (50-genes), aCGH	Alterations with known/predicted pathogenicity available in clinical trials or off-label, compassionate use	40% (*n* = 58)	Children, high-risk/relapsed/refractory solid tumors	14–15	[24]
Peds-MiOncoSeq	Tumor + normal WES, RNAseq	Alterations which altered diagnosis, risk stratification or treatment, or diagnosed a cancer predisposition syndrome	46% (*n* = 91)	≤ 22 y, refractory/rare cancer	12–14	[29]
iCAT	Sequenom assay (47 genes) or NGS (275 genes), aCGH	Alterations which altered diagnosis or treatment, or diagnosed a cancer predisposition syndrome	48% (*n* = 89)	≤ 30 y, recurrent, refractory, or high-risk extracranial solid tumor	12–14	[30]
INFORM	Tumor + normal WES, WGS, RNAseq, aCGH, methylation	Theoretically targetable by an approved drug or an investigational agent, either directly or indirectly in the affected pathway	86% (*n* = 519)	1–40 y, relapsed/refractory or some primary cancer	15–19	[20]
MOSCATO-01	aCGH, NGS (75 genes), WES, RNAseq	A molecular alteration or affected pathway theoretically directly/indirectly targetable by an approved or developmental drug	61% (*n* = 69)	≤ 24 y, incurable, relapsed or resistant solid malignancy	12–16	[31]
Spanish initiative	Tumor + normal NGS (50 or 159 genes)	An alteration with biological activity that could be targeted with a therapy already used in vivo	15% (*n* = 84)	Children/adolescents, relapsed/refractory solid tumors	14	[32]
FM Experience	NGS (236 genes)	At least one alteration targeted by a therapeutic on the market or in clinical trials	60% (*n* = 400)	≤ 21 y, solid tumors, leukemias	14	[33]
Rady Children’s Hospital	Tumor NGS (315 or 405 genes)	Mutations which altered diagnosis or treatment, or diagnosed a cancer predisposition syndrome	74% (*n* = 57)	≤ 21 y, neuro-oncologic malignancies	14–16	[34]
UCSC Treehouse	Tumor WGS, WES, NGS, RNAseq	NCI Pediatric MATCH study considerations were used	69% (*n* = 144)	≤ 29 y, cancer	16–17	[35]
Pediatric MATCH	Tumor DNA and RNA—NGS panel	Actionable mutations based upon available clinical and pre-clinical data for at least one of the 10 treatment arms	32% (*n* = 1000)	≤ 21 y, refractory/recurrent tumors	17–22	[21]
SMC	Tumor NGS (381 genes)	Targetable variants with FDA-approved drugs, drugs available in other countries for use or in clinical trials	38% (*n* = 53)	≤ 21 y, refractory/relapsed solid tumors	16–18	[14]
Zero Childhood Cancer Program	WGS, RNAseq, methylation array	Alterations with therapeutic targets	71% (*n* = 247)	≤ 31 y, high-risk malignancies	15–19	[15]
TRICEPS	WES, RNAseq	Potentially actionable: alteration is a target or negative biomarker of an approved or developmental drug	87% (*n* = 62)	≤ 21 y, relapsed/refractory or hard-to-treat cancer	14–18	[16]
MAPPYACTS	WES, RNAseq	Genetic alteration that could represent a potential therapeutic target	69% (*n* = 436)	≤ 18 y, recurrent/refractory malignancy	16–20	[19]
POB of NCI	Tumor + normal WES, RNAseq	Targetable variants with approved or developmental drugs, reportable germline mutation, or a change of diagnosis	51% (*n* = 59)	≤ 25 y, non-central nervous system solid tumor	10–14	[18]
G4K	Tumor + normal WGS, WES, RNAseq	(Likely) pathogenic and targetable by an FDA-approved drug or an investigational agent, directly or its downstream pathway	81% (*n* = 253)	Newly diagnosed/relapsed/refractory pediatric cancers, unselected for tumor type	15–17	[22]
iTHER	WES, WGS, RNAseq + array, methylation array	On-/off-label, clinical trial, compassionate use	59% (*n* = 80)	< 30 y, relapsed/refractory tumor, no standard protocol	17–20	[23]
GAIN/iCAT2	NGS, RNAseq	Variant for which an iCat therapeutic recommendation was made	69% (*n* = 345)	≤ 30 y, relapsed/refractory or high-risk extracranial solid tumors	15–19	[25]
AflacPM1702 (APMP)	Tumor + normal WES, tumor RNAseq	Alteration used to inform Clinical management (linked to an FDA-approved drug or drugs in preclinical testing or early phase clinical trials	65% (*n* = 126)	≤ 30 y pediatric brain tumors, hematologic malignancies, and extracranial solid tumors	18–22	[26]
PROFYLE^b^	Tumor + normal genomics and transcriptomics	Potentially actionable	40% (*n* > 900)	≤ 29 y, hard-to-cure cancer	16–22	[27]

*BASIC3* Baylor College of Medicine Advancing Sequencing in Childhood Cancer Care, *PIPseq* Precision in Pediatric Sequencing Program, *SMPAEDS* Stratified Medicine Paediatrics, *MBB* Molecular Biology Tumor Board, *Peds-MiOncoSeq* Pediatric Michigan Oncology Sequencing, *iCAT* Individualized Cancer Therapy, *INFORM* Individualized Therapy for Relapsed Malignancies in Childhood, *MOSCATO* Molecular Screening for Cancer Treatment Optimization, *FM* Foundation Medicine, *UCSC* University of California, Santa Cruz, *MATCH* Molecular Analysis for Therapy Choice, *SMC* Samsung Medical Center, *TRICEPS* Personalized Targeted Therapy in Refractory or Relapsed Cancer in Childhood, *MAPPYACTS* Molecular Profiling for Pediatric and Young Adult Cancer Treatment Stratification, *POB* Pediatric Oncology Branch, *NCI* National Cancer Institute, *G4K* Genomes for Kids, *iTHER* Individualized Therapies, *GAIN* Genomic Assessment Informs Novel Therapy Consortium, *APMP* Aflac Precision Medicine Program, *PROFYLE* Precision Oncology For Young People, *POG* Personalized Oncogenomics, *KiCS* SickKids Cancer Sequencing, *WES* whole-exome sequencing, *RNAseq* RNA-sequencing, *NGS* next-generation sequencing, *WGS* whole-genome sequencing, *aCGH* microarray-based comparative genomic hybridization, *OncoKB* Precision Oncology Knowledge Base, *COSMIC* Catalogue of Somatic Mutations in Cancer, *SNV* single nucleotide variants, *CNV* copy number variations, *FDA* Food and Drug Administration

^a^Actionable findings per successful sequencing

^b^PROFYLE was designed to unite and build upon three programs POG, KiCS, and TRICEPS

The general method of interpretation of molecular profiles is matching each genetic alteration to targeted therapies one by one and ranking them based on the highest-level evidence [41]. Matching therapies one by one, without considering the whole molecular profile, failed to provide meaningful clinical benefit on a pool of solid tumors [42]. However, treatment options with high evidence levels can provide clinical benefit in some adult cancer types [5, 43] and in pediatric cancers [19, 20]. The limitation of this simplistic approach is that most cancers are driven by a complexity of multiple driver alterations, making standardized decisions difficult [44]. Two molecular tumor boards (MTBs), located in the same country, were shown to have an agreement rate of just 44% on high-dimensional data [45]. Another possibility is to use combination therapies to match more than half of drivers. “Matching Score”, the ratio of drivers targeted, has been shown to correlate with outcome in the I-PREDICT clinical trial [2], indicating the need for complex matching algorithms for therapy planning. This method lacks ranking of driver alterations to select therapies matching the most important drivers and associated indirect targets. Moreover, in many cases, there is no targeted therapy available for all drivers, or the use of combination therapies is limited by toxicity or cost.

Recently, we proposed a new algorithm, the digital drug assignment (DDA), to identify targeted therapies that target the most important targets of the most important drivers based on a calculated cumulative score, the aggregated evidence level (AEL). This algorithm considers the numerous direct and indirect relationships between multiple driver genes with the same targeted therapies. DDA has been implemented into a software system and validated on data from the SHIVA01 clinical trial [46]. The analysis found a significant correlation between clinical benefit and AEL scores, implying that DDA can be a useful method to choose between genomic information-based treatment options.

The frequency of specific driver alterations can be profoundly different in pediatric cancers compared to adult solid tumors analyzed in SHIVA01. Therefore, in this study, we aimed to collect real-world evidence about the clinical utility of computational decision support using DDA in pediatric cancer. The digital nature of computational interpretation enabled us to analyze the performance of the system on pediatric profiling data in different terms [ratio of actionability, resistance prediction, correlation with the European Society for Medical Oncology scale for clinical actionability of molecular targets (ESCAT) criteria] and virtually compare the potential utility of different gene sets covered by commercial panels. Finally, we analyzed the correlation of absolute and relative AEL scores with tumor board decisions.

## Methods

### Patients

Between 2017 and 2020, 103 patients (< 21 years) from multiple Hungarian pediatric oncology institutes were included in the precision oncology program. Patients were selected for this study by a tumor board including an oncologist, a genetic counselor, medical doctors, and molecular biologists. Selection criteria for high-risk patients included (1) having malignancy with Eastern Cooperative Oncology Group criteria (ECOG) 0–2; (2) relapsed/refractory disease or poor prognosis at diagnosis; (3) receiving an internationally accepted first-line treatment containing bone marrow transplantation and/or (4) having a prognosis of < 50% overall survival.

Participants or their parents signed consent for data analysis after risks and benefits had been explained. Consents extend to the purposes of the study, the limitations of the tests and the understanding and approval of data storage, data analysis and management. Ethics approval was obtained from the National Institute of Pharmacy and Nutrition (OGYÉI/50268/2017) before conducting the study. Treatment of patients was the decision of the treating physician, and follow-up was not part of the study.

### Process of the precision oncology program

MTB reviewed available samples, ranked them for molecular testing and decided on complementary tests. Tissue samples were tested by WES, immunohistochemistry (IHC), microsatellite instability (MSI) analysis and fluorescent in situ hybridization (FISH). In case samples were unfit for WES, a smaller targeted sequencing (591-genes or 58-genes, or Sanger sequencing) was performed. After bioinformatic filtering, molecular profiles were evaluated by the DDA system. Finally, the MTB reviewed the results to provide final therapy recommendations for the treating physician. More information on the precision oncology process can be found on the webpage of the system [47].

### Sample preparation

Molecular diagnostic tests were performed on the available formalin-fixed paraffin-embedded (FFPE) tumor samples obtained during routine procedures (biopsy, surgery). All tumor specimens were reviewed by a molecular pathologist who determined the percentage of tumor nuclei and adequacy for profiling. A tumor/normal cell ratio of 10% was required for single nucleotide variants (SNV) detection and 30% for copy number variations (CNV) analysis. A minimum of 60 tumor cells in the sample slices were accepted for IHC and FISH analysis.

Totally 10 6 µm-thick unstained slices containing tumor cells were required for DNA extraction after pathological validation. Specimens decalcified by ethylene diamine tetraacetic acid (EDTA) were also considered adequate for molecular profiling. DNA was extracted from FFPE samples according to standard procedures using a QIAamp DNA FFPE Tissue Kit (Qiagen, 56404). DNA quantity and quality were determined with a NanoDrop (NanoDrop Technologies, D439) and by PCR. A minimum of 1000 ng of total DNA was used for library preparation.

### Sequencing analysis

Exome sequencing was performed at two providers (Eurofins Genomics and MedGenome). The laboratory-developed tests used general purpose reagents and the Agilent SureSelect Human All Exon V5/V6 capture kit for library generation. WES was performed to an average 100–120X depth on the HiSeq2500 using paired-end (2 × 125 bp) sequencing followed by data processing.

Sequence cleaning was performed by removing adapter sequence bases with low quality from the 3′ and 5′ ends, bases that had an average phred quality below 15 and clipped reads shorter than 36 bp. Mapping to the reference sequence GRCh37 (hg19) was carried out using Burrows–Wheeler Aligner (BWA) with default parameters. Only uniquely mapped on-target reads were processed further. Reads were deduplicated using Sambamba to remove the artificial coverage caused by the PCR amplification step during library preparation and/or sequencing.

SNP and insert and deletion (InDel) calling was performed using GATK’s Haplotype Caller. Variants detected were annotated based on their gene context using SNPeffect Metrics that are used to evaluate the quality of a variant are annotated using GATK’s Variant Annotator module.

### Copy number variation

CNVs were detected using the software package CNVkit, which uses normalized read depths to infer copy numbers evenly across the exome/genome. CNVkit uses both the on-target reads and the nonspecifically captured off-target reads to calculate log2 copy ratios across the genome for each sample. The on- and off-target read depths are combined, normalized to a reference derived from control samples, and corrected for several systematic biases (GC content, sequence complexity and targets) to result in a final table of log2 copy ratios. The segmentation algorithm uses log2 ratio values to infer discrete copy number events. Copy number events with a minimum 100 × coverage were reported in samples with a tumor cell ratio of at least 30%.

### Bioinformatic analysis of next-generation sequencing results

Sequencing output files were processed by a laboratory-developed filtering process that integrated bioinformatic software such as Ingenuity Variants Analysis or VarSome Clinical. The Integrative Genomics Viewer (IGV) visualization tool was used to check candidate variants and their genomic neighborhood. A depth of more than 20 reads was required to determine a variant, with quality thresholds of 20 and allele frequency of 1% in the alternate allele. Somatic mutations were enriched by filtering out variants with at least 10% in databases of 1000 Genomes, the Exome Aggregation Consortium (ExAC) or National Heart, Lung, and Blood Institute GO Exome Sequencing Project (NHLBI ESP) exomes. Variants classified as “benign” or “likely benign” by American College of Medical Genetics and Genomics (ACMG) were also excluded. Our filtering protocol includes a virtual panel of nearly 1000 genes linked to tumorigenesis in literature data and our evidence database.

### Additional molecular diagnostic tests

Fluorescent PCR-based fragment analysis (MSI Analysis System, Version 1.2, Promega, MD1641) was used to identify microsatellite instability. Examination of the length of five known gene sequences containing mononucleotide repeats by capillary electrophoresis identified the presence of instability indicative of a defect in DNA repair enzymes (mismatch repair). Two categories of MSI status MSI-high (MSI-H) and microsatellite stability (MSS) were distinguished depending on showing more or less than 20% instability of five mononucleotide repeats (NR-21, NR-24, BAT-25, BAT-26, MONO-27).

FISH by the ZytoLight Direct Label system (ZytoVision GmbH, Z-2028-20) was used to detect genetic aberrations of translocations (*ALK*, *RET*, *ROS1*), amplifications (*EGFR*, *HER2*, *MET*, *FGFR1*, *PIK3CA*), and chromosomal aneuploidies. Hybridization images of fluorescently dual-labeled probes to the target regions were scanned by a Pannoramic MIDI Scanner and visualized with Pannoramic Viewer^™^ software (3DHISTECH). The results were interpreted according to American Society of Clinical Oncology (ASCO) consensus guidelines and University of Colorado Cancer Center (UCCC)-Cappuzzo’s criteria, updated from the latest published data.

IHC tests were performed to evaluate the expression of programmed cell death 1 ligand 1 (PD-L1) (22C3 pharmDx, DAKO, M365329-2), human epidermal growth factor receptor 2 (HER2) (anti-HER-2/neu (4B5) rabbit monoclonal primary antibody, Ventana, 790-2991) and translocation of anaplastic lymphoma kinase (ALK) (FLEX monoclonal mouse anti-human CD246, DAKO, IS641). Sample preparation and staining were performed using the DAKO EnVision FLEX system.

If an important germline alteration was suspected, the mutation was analyzed by Sanger sequencing on a blood or saliva sample of the patient. A bidirectional sequence was assembled and aligned to the reference gene sequence based on the human genome build GRCh37/hg19.

### Biomedical interpretation with the digital drug assignment-based software system

The DDA-based software system used in this study was the Realtime Oncology Treatment Calculator v1.28-1.66 (Genomate Health Inc) [46]. First, the evidence database of the DDA-based software system was updated regarding all variants of the patients’ molecular profiles through a manual search. In addition to scientific publications, databases used to assess the clinical relevance of variants were the following: Catalog of Somatic Mutations in Cancer (COSMIC), National Center for Biotechnology Information (NCBI) database of single nucleotide polymorphisms (dbSNP), NCBI ClinVar, SNPEffect, International Agency for Research on Cancer (IARC), Breast Cancer Information Core (BIC), and UniProt.

The DDA system is a rule-based knowledge engine capable of classifying genetic alterations, and prioritizing target molecules and compounds based on the related evidence database and its proprietary algorithm. A detailed description of DDA has been published previously [46]. In brief, the evidence database contains parameterized scientific data about the oncogenicity and targetability of molecular alterations. Classification and ranking are based on scoring the evidence items referring to associations between the patient’s alterations, targets and/or compounds. The evidence scores are calculated based on parameters about data reliability (e.g., clinical or preclinical study, publication type), weighted according to their relevance in the case (e.g., same or different tumor type, same mutation or data about the gene in general) and summed for each alteration, target and compound. The weighted sum generates the AEL. In this way, automated prioritization incorporates multiple data sources, preclinical and clinical results, and even conflicting evidence while also taking resistance mechanisms into account. Reports in 2017 were generated manually; therefore, for easier analysis, DDA was issued on these cases in 2020. However, we kept the original recommendations.

Next, a report containing DDA results and text summaries about the pre/clinical actionability of all driver alterations was generated and submitted for MTB discussion.

### Expert opinion, data transfer to treating physician

MTB meetings were held every working day. The role of the MTB was to align DDA results with patient characteristics, performance status, previous therapies, potential combination therapies, toxicities, and drug availability. Reports and drug recommendations were issued to the treating physician for further use.

### Classification of digital drug assignment-based treatment recommendations according to the ESCAT evidence scale

Data exported from the DDA-based software system for each case were used for descriptive statistics in Excel. ESCAT reanalysis was performed using all pieces of evidence included in the calculations along the following guidelines: ESCAT I—approved, biomarker-based compounds in the same indication, ESCAT II—clinical, biomarker-based evidence for a compound in the same tumor type, ESCAT III—clinical, biomarker-based evidence for a compound in another tumor type, ESCAT IV—preclinical, biomarker-based evidence or indirect preclinical evidence.

### Performance analysis based on different gene panels

The list of genes covered by different panels was downloaded from the vendor’s website. WES variants were filtered for genes of different panels; actionability/resistance rates were defined, and it was also checked whether the top-ranked driver would have been covered by the panel. Actionability means that a driver or variant of uncertain significance (VUS) in a driver gene is targetable with a registered targeted therapy, and if such an alteration was detected, the case was considered actionable. The number of cases with actionable findings per the number of cases with successful sequencing gives the actionability rate. The top-ranked driver is the one with the highest AEL score. It has also been assessed if filtering would have affected therapy selection. The MTB decision was considered altered when the gene panel did not cover the driver that the original MTB decision was built on. Standard therapy recommendations were not affected by downsampling.

### Statistical analysis

Graphs were generated in Excel and GraphPad Prism 9. Statistical analysis, where applicable, was carried out in Prism.

## Results

### Patient characteristics

**Fig. 1:**
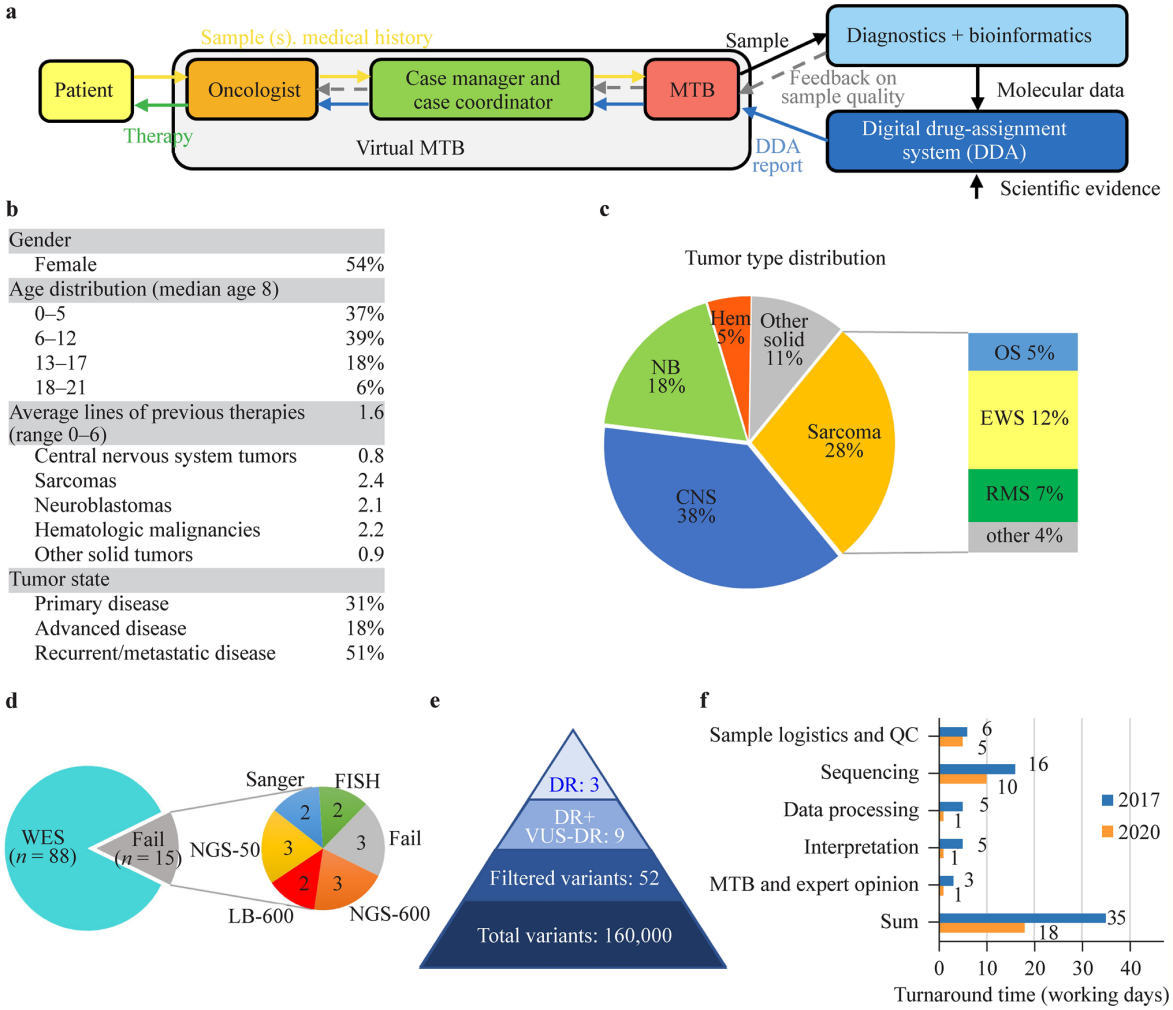
Process, patient characteristics and analysis. **a** Schematic representation of the workflow of the precision oncology program. Inclusion was mostly initiated by oncologists. An expert case manager and a case coordinator were assigned to each case to collect all the necessary information. The oncologist and the case manager presented the data, medical history, and samples to the molecular tumor board (MTB), where a decision on testing and samples was made. After diagnostics and bioinformatic analysis, a DDA report was generated and discussed with the MTB for final therapy recommendation. **b** Patient characteristics. **c** Tumor type distribution of patients. **d** Representation of molecular profiling types performed. **e** Average variant counts per sample identified by WES or a 591-gene panel analysis. **f** Turnaround time improvement between 2017 and 2020 split by process steps. *DDA* digital drug assignment, *WES* whole-exome, *QC* quality check, *NB* neuroblastoma, *CNS* central nervous system tumors, *Hem* hematological cancers, *EWS* Ewing sarcoma, *RMS* rhabdomyosarcoma, *OS* osteosarcoma, *NGS-600* next-generation sequencing of a 600-gene panel, *NGS-50* next-generation sequencing of a 50-gene panel, *LB-600* next-generation sequencing of a 600-gene panel starting from liquid biopsy*, DR* driver, *VUS-DR* variant of unknown significance in a driver gene

Between 2017 and 2020, 103 patients (< 21 years) from multiple pediatric oncology centers in Hungary were included in the precision oncology program of our clinical practice (Fig. 1a). Inclusion was defined by clinical necessity, and primarily patients with high-risk, refractory, or relapsed childhood cancer were recruited. Patient characteristics are detailed in Fig. 1b and Supplementary Table 1. Patients presented with various disease stages: newly diagnosed localized disease (31%), advanced disease progressed on previous treatment (18%), recurrent or metastatic disease (51%), and were diagnosed with a diverse representation of malignancies, including central nervous system (CNS) tumors (38%), sarcomas (28%), neuroblastomas (18%), other solid tumors (11%), and hematologic malignancies (5%) (Fig. 1c).

A multidisciplinary MTB reviewed available samples, ranked them based on adequacy for molecular testing, and decided on complementary tests. Tissue samples were analyzed by WES, IHC, MSI testing, and FISH. Non-NGS molecular diagnostic tests yielded positive results in six patients. Amplification was detected by FISH analysis in five cases (of 79, 6%, up to seven genes tested) of the following genes: *ERBB2* (× 2), *FGFR1*, *MET*, *and PIK3CA*. One of 73 samples showed PD-L1 positivity, and none of 77 samples were MSI-high (Supplementary Table 1).

WES results were obtained in 88 cases; samples from 15 patients were unfit for WES. For these patients, targeted analysis was performed based on sample adequacy, namely, 591-gene NGS (*n* = 3), 58-gene NGS (*n* = 3), 591-gene NGS from liquid biopsy (*n* = 2), multiple single-gene Sanger sequencing (*n* = 2), or FISH analysis only (*n* = 2) (Fig. 1d). Samples from three patients did not yield any information, resulting in a total of 100 patients with molecular data.

After bioinformatic filtering, on average, 52 variants were identified with WES or 591-gene NGS. All test results were uploaded to the DDA-based software system, which provided a report ranking molecular alterations, associated targets, and compounds based on the updated evidence database. The system identified on average three driver alterations and six variants of unknown significance in a driver gene per case (Fig. 1e). Finally, the MTB reviewed the results to provide therapy recommendations for the treating physician.

Through technological developments, the turnaround time of the entire process was reduced from 35 to 18 working days over the 4 years of the study, with the interpretation part requiring less than 1 day due to the semiautomated drug-assignment system (Fig. 1f). The sequencing time decreased due to technical progress, while data processing became faster thanks to the development of bioinformatic systems. The improvement of the database and algorithm and the reduction of manual work led to reduced interpretation time. Advanced logistics and virtuality contributed to an accelerated MTB process.

### Actionability of digital drug assignment in pediatric cancers

**Fig. 2:**
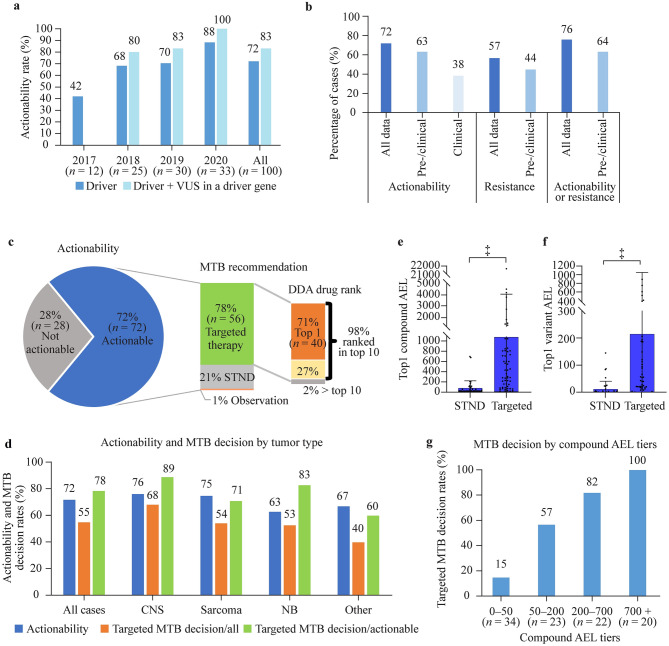
Relationship between DDA-identified actionability and MTB decisions. **a** Actionability ratios of all cases. A molecular profile was considered actionable when at least one driver (or VUS in a driver gene) was targetable with a registered drug (not limited to approved indications, but developmental compounds excluded). **b** Actionability and resistance rates filtered by evidence types. Resistance was identified when at least one registered drug was negatively associated with a driver in the molecular profile. **c** MTB decision and DDA drug ranks. DDA drug rank is the rank score of compounds ordered by aggregated evidence level (highest level obtains rank 1). **d** Actionability rates and MTB targeted therapy decision rates by tumor type. **e** and **f** First-ranked (maximum) compound and variant AEL by MTB decision. Mean + SD, ^‡^*P* < 0.001, two-tailed Mann–Whitney *U* test. **g** Association of MTB targeted therapy decision rates (targeted/actionable cases) with highest compound AEL tiers, *P* < 0.001, Chi-square test. The total case number is 99 due to the exclusion of a case with no treatment (only observation) recommended. *DDA* digital drug assignment, *MTB* molecular tumor board, *VUS* variant of unknown significance, *STND* standard therapy, *CNS* central nervous system, *NB* neuroblastoma, *AEL* aggregated evidence level, *STND* standard therapy, *SD* standard deviation. ^‡^*P* < 0.001

The DDA-based software system deployed an average of 427 scientific publications and a network of 1212 associations for the analysis of each molecular profile. The system identified matching registered (on-/off-label) targeted treatment options in 72% of the 100 patients with molecular diagnostic results (same ratio with WES only, 63/88). With a rising tendency, actionability culminated at 88% in 2020 due to the continuous expansion of approved drugs and real-time updates of the evidence database (Fig. 2a). When driver genes with variants of unknown significance are included in the calculation, the overall actionability is 83%. Previous studies reported actionability rates between 15 and 87%; however, most of them considered developmental compounds as well when determining actionability (Table 1). Our results indicate that a substantial proportion of high-risk pediatric solid tumors have actionable alterations according to the DDA.

The actionability rates were also analyzed considering clinical or clinical and preclinical (pre/clinical) data only for the definition of drivers (not considering drivers with in silico and frequency-type data only) while keeping the registered drug criterion. Actionability rates were 63% and 38% based on pre/clinical and clinical evidence, respectively (Fig. 2b). Although important, information on the discovered potential resistance mechanism to molecularly targeted agents is mostly underdiscussed in previous studies. In our study, resistance to a registered drug was identified in 57% of patients, with a pre/clinical evidence-based resistance rate of 44% (Fig. 2b). Altogether, actionability or resistance has been revealed in 76% of patients, with 64% based solely on pre/clinical evidence (Fig. 2b). We identified on average 11.5 registered drugs positively or negatively associated with each WES profile.

### Concordance between digital drug assignment scores and MTB decisions

After DDA-based report generation, a multidisciplinary MTB discussed therapy recommendations within 1–2 days. The role of the MTB is to align DDA results with patient characteristics, performance status, previous therapies, potential combination therapies, toxicities, and drug availability [48–51]. In total, MTB supported the use of a molecularly targeted agent in 55 cases (55%). MTB approved the future use of a targeted compound in 78% (56/72) of cases considered actionable (Fig. 2c). Of these, 55 (98%) were ranked among the top 10 registered compounds, and 40 (71%) were ranked the 1st by the DDA system, simplifying targeted MTB decisions. The most frequently recommended top-ranked compound was olaparib (*n* = 6), followed by selumetinib and ruxolitinib (*n* = 3 each), erdafitinib, cetuximab, palbociclib, imatinib, trastuzumab (*n* = 2 each), and a wide variety of other approved targeted agents (Supplementary Table 1), highlighting the diversity of individual tumors. Actionability values and MTB-targeted decision rates in different tumor types are depicted in Fig. 2d, with the highest actionability and targeted decision rates in CNS tumors.

During DDA, a quantitative score, the aggregated evidence level (AEL), is assigned to each driver and compound by the algorithm. AEL represents the number, scientific impact, and clinical relevance of evidence relations (pieces of cancer-related scientific information) in the system, connecting tumor types, molecular alterations, targets, and compounds and provides the basis for ranking. We evaluated how MTB decision correlates with the AEL scores of top-ranked variants and drugs; therefore, we split cases by MTB decision (standard-of-care or targeted therapy). In the targeted therapy group, AELs of top-ranked alterations and drugs were significantly higher than in the group of patients receiving molecular profile-independent therapy (Fig. 2e, f). Accordingly, splitting the cases into four AEL groups of similar population density revealed that the ratio of targeted MTB decisions increased with drug AEL (Fig. 2g). Together, these data suggest that the DDA-based software system can be a useful tool for driver and compound ranking as an input for the MTB both in decisions of targeted versus chemotherapy and choosing between targeted treatment options.

### Comparative analysis of digital drug assignment and ESCAT-based treatment recommendations

**Fig. 3:**
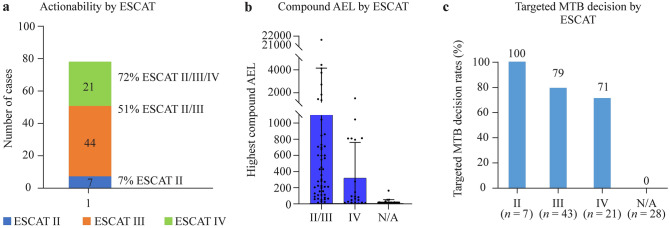
Comparative analysis of DDA and ESCAT-based treatment recommendations. **a** Actionability rates and case numbers by ESCAT tiers. **b** Highest compound AEL scores by ESCAT tiers. Mean + SD. **c** Targeted therapy MTB decision rates by ESCAT tiers. The total case number is 99 due to the exclusion of a case with no treatment (only observation) recommended. *DDA* digital drug assignment, *ESCAT* European Society for Medical Oncology scale for clinical actionability of molecular targets, *AEL* aggregated evidence level, *SD* standard deviation, *MTB* molecular tumor board

Although DDA is not based on ESCAT categories, storing all structured pieces of evidence used for the automatic calculations enabled us to retrospectively analyze cases to classify treatment options based on ESCAT criteria. We identified 7 cases with ESCAT II, 44 with ESCAT III, and 21 with ESCAT IV evidence, leading to 7% actionability by ESCAT II, 51% by ESCAT II/III, and 72% by ESCAT II/III/IV categories. No ESCAT I evidence was identified, given that no samples had MSI/high tumor mutational burden (TMB) or neurotrophin receptor kinase (NTRK)-rearranged status (Fig. 3a).

To determine how AEL values relate to ESCAT categories, we plotted AEL by ESCAT categories. Since tier II contained only seven elements, we merged it with tier III. As shown in Fig. 3b, AEL values on average are higher at higher (II/III) than in the lower (IV) ESCAT category and the lowest when no ESCAT category was applicable (no driver or targeted agent available), although there is not a strong concordance due to the deviation of AEL in the different categories. MTB targeted decision rates also follow the rank of ESCAT categories, but it is important to mention that even in Tier IV with strong preclinical or indirect evidence, we had a targeted decision rate of over 70% (Fig. 3c).

### Actionability of digital drug assignment based on different gene sets

**Fig. 4:**
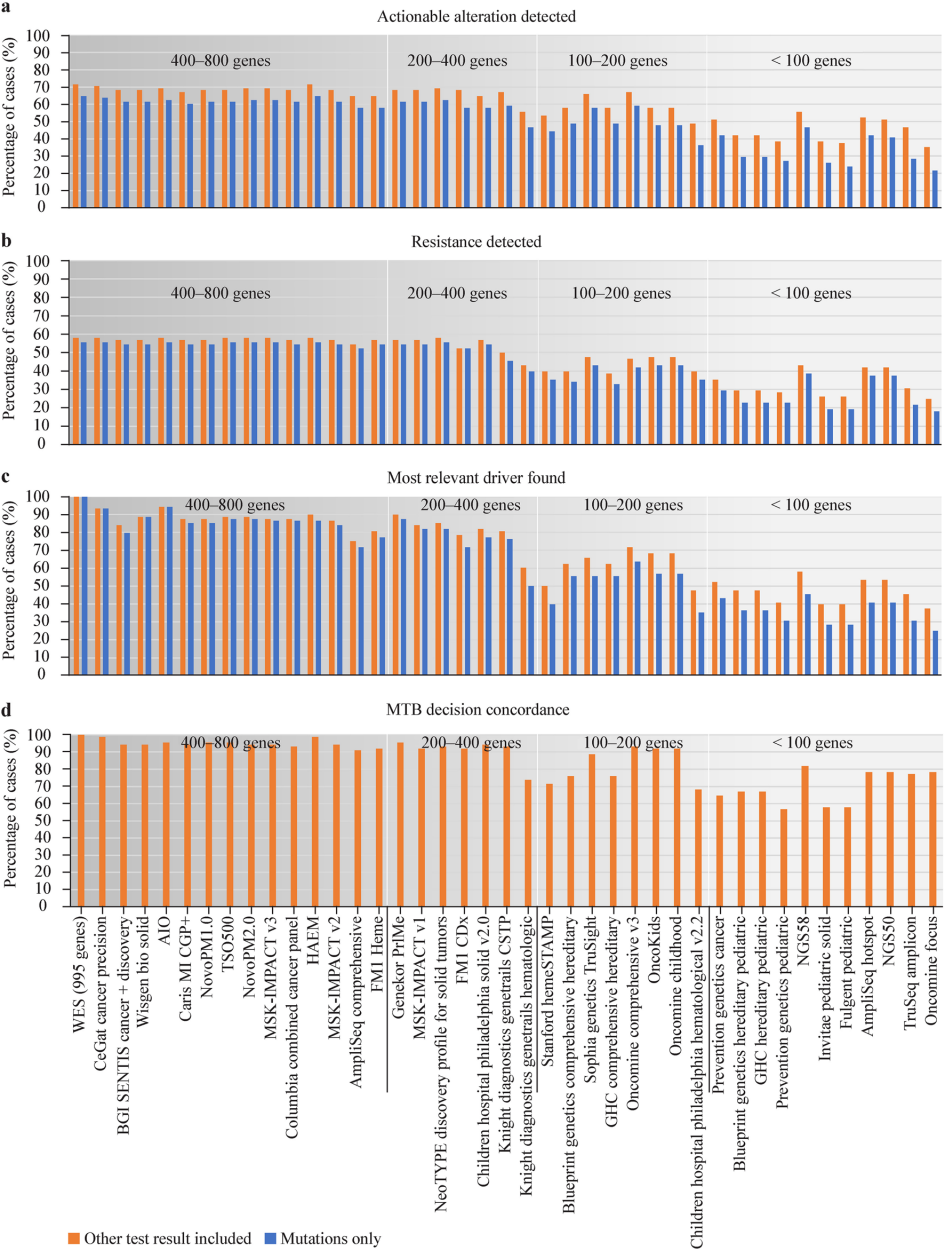
Reanalysis of WES-based results by DDA and MTB based on different gene sets (*n* = 88). **a** Actionability and **b** resistance rates by gene sets included in the selected panels. **c** Most relevant driver covered by panels compared to WES-995-gene analysis (100%). **d** MTB decision concordance by panels compared to WES-995-gene analysis (100%). The MTB decision was considered altered when the gene panel did not cover the driver that the original MTB decision was built on. Standard therapy recommendations (*n* = 39) were not affected by downsampling. Orange bars—additional test results considered (IHC, MSI, FISH); blue bars—NGS results only. Panel names with gene lists are included in Supplementary table 1. *WES* whole-exome sequencing, *DDA* digital drug assignment, *MTB* molecular tumor board, *BGI* Beijing Genomics Institute, *AIO* former INVIEW Oncopanel All-in-one by GATC, current name INVIEW Oncoprofiling (591 genes), *MI CGP* + Molecular Intelligence^®^ Comprehensive Genomic Profiling PLUS, *NovoPM* Novogene Precision Medicine, *TSO* TruSight Oncology, *MSK-IMPACT* Memorial Sloan Kettering Integrated Molecular Profiling of Actionable Cancer Targets, *HAEM* Hemato-oncology enrichment panel, *FM1* FoundationOne, *PrIMe* Precision Individualized Medicine, *CDx* companion diagnostic, *CSTP* Comprehensive Solid Tumor Panel, *HemeSTAMP* Stanford Tumor Actionable Mutation Panel for Hematopoietic and Lymphoid Neoplasms, *NGS* next generation sequencing

Although many publications support the necessity of the most comprehensive molecular profiling in pediatric tumors [12, 19, 20, 26], WES is still not available everywhere. In many cancer centers, only single genes or an ~ 50-gene NGS hotspot panel (designed for adult malignancies) is applied. Even when WES/WGS is utilized, variant interpretation is normally restricted to a virtual panel of ~ 6–700 genes associated with cancer. In our analysis, we used a comprehensive 995-gene list containing all COSMIC cancer census genes and other manually selected genes that have been implicated in adult or pediatric cancer (Supplementary Table 1). To provide an approximation of panel sequencing performance for our WES analyses (*n* = 88), we analyzed the virtual coverage of the results by commercially available NGS panels (Fig. 4). In one approach, we hypothesized that all non-NGS-based test results (FISH, IHC, MSI) are also obtained in addition to panel sequencing; in the other approach, we focused only on mutations from NGS results.

In terms of overall actionability and resistance identification, DDA performed similarly well on 400–800-gene panels compared to the virtual 995-gene panel based on WES (Fig. 4a, b). However, these panels covered the strongest driver according to the DDA only in around 90% of the cases (Fig. 4c). This means that in this virtual analysis, the strongest driver would not have been found with 400–800-gene panels in 10% of the patients, also affecting targeted decisions. Most panels down to 200 genes detected > 80% of the strongest drivers, but panels below 200 genes delivered significantly different results, profoundly compromising treatment decisions (Fig. 4d). Taken together, we can provide a performance estimation of DDA-based decision support based on different commercial panels compared to a virtual 995-gene panel of WES in pediatric tumors.

### Clinically relevant driver alterations identified by digital drug assignment in different pediatric tumor types

**Fig. 5:**
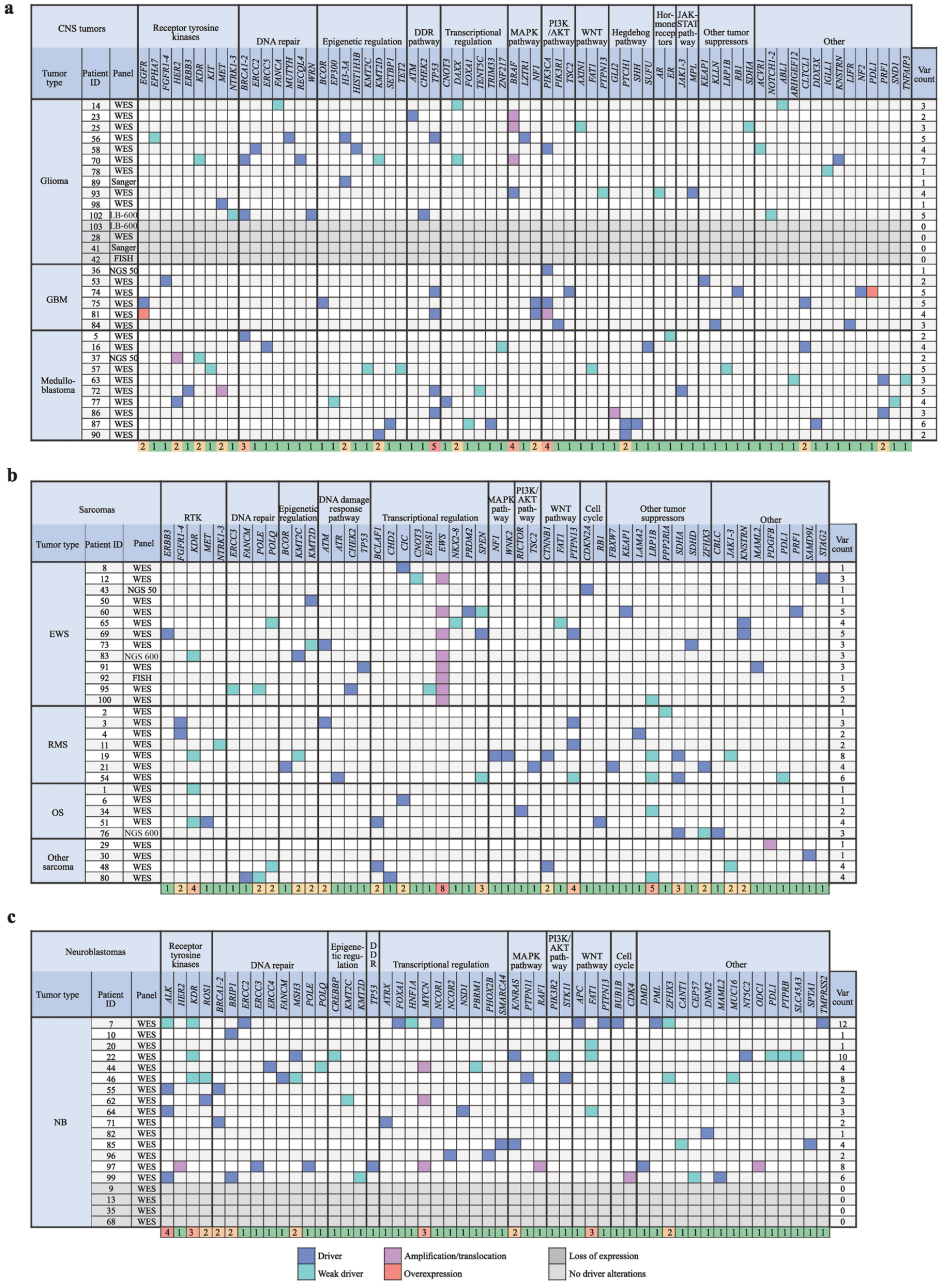
Clinically relevant driver alterations identified by tumor type. Altered genes and their pathways in **a** central nervous system (CNS) tumors, **b** sarcomas, and **c** neuroblastomas (NB). Different alteration types are color coded. The last row of each table shows gene alteration counts in the population studied, heatmap-colored. *WES* whole-exome sequencing, *NGS* next-generation sequencing, *NGS-600* next-generation sequencing of a 600-gene panel, *NGS-50* next-generation sequencing of a 50-gene panel, *LB-600* next-generation sequencing of a 600-gene panel starting from liquid biopsy, *FISH* fluorescent in situ hybridization, *GBM* glioblastoma multiforme, *RTK* receptor tyrosine kinases, *DDR* DNA damage response, *EWS* Ewing sarcoma, *OS* osteosarcoma, *NB* neuroblastoma, *RMS* rhabdomyosarcoma, *var* variation, *MAPK* mitogen-activated protein kinase, *PI3K* phosphatidylinositol-4,5-bisphosphate 3-kinase, *JAK* Janus kinase, *STAT* signal transducer and activator of transcription

To present the detected driver alterations of the three most prevalent tumor groups (CNS tumors, neuroblastomas, and sarcomas), we generated a table from the affected genes in each group (Fig. 5, with full list of alterations in Supplementary Table 1). Alterations in receptor tyrosine kinases, DNA repair genes, and genes involved in epigenetic regulation were found in all three subgroups (glioma, glioblastoma multiforme, and medulloblastoma) of CNS tumors (Fig. 5a). Hedgehog gene alterations were characteristic of medulloblastoma, mitogen-activated protein kinase (MAPK) and phosphatidylinositol-4,5-bisphosphate 3-kinase (PI3K)/Akt pathway alterations were typical in glioma and glioblastoma, and DNA repair gene involvement was detected in glioma and medulloblastoma. Sarcomas showed a more diverse landscape of gene alterations (Fig. 5b). Interestingly, Ewing sarcomas did not present alterations in MAPK or PI3K/Akt pathway genes. In neuroblastomas, DNA repair genes and players in transcriptional regulation were mostly altered in addition to ALK and N-MYC (Fig. 5c). Notably, we detected proven driver mutations (AEL > 20) in the following untargetable genes: *DICER1, MC1R, KNSTRN, DDX3X, ERCC3,* and *NT5C2*.

In cases when a germline alteration or a genetically encoded disease was suspected, we performed targeted sequencing of patients (and parents when applicable) in search of the alteration in normal tissue. In two suspected cases with *DICER1* mutations, disease inheritance was excluded for the relief of parents and siblings. Importantly, germline results even changed the diagnosis of a patient to a noncancerous genetic disorder [fibrodysplasia ossificans progressiva (FOP), *ACVR1* p. R258S] [52], and chemotherapy was discontinued immediately. Taken together, our data can also provide valuable insight into the gene alterations of pediatric tumors.

## Discussion

In this report, we reviewed the first 100 cases evaluated in our pediatric precision oncology program involving patients from different institutes. Our results indicate that DDA-based decision support using WES is feasible and demonstrates clinical utility in pediatric malignancies. We show that a substantial proportion, over two-thirds, of high-risk solid tumors have clinically impactful actionable alterations and that there are other clinical utilities of comprehensive sequencing. The high percentage of targeted therapy recommendations by the MTB in the actionable subgroup (77%), of which 78% were top-ranked by the DDA-based computational tool, and the correlation of top-ranked drug AEL values with targeted MTB decisions provide evidence that DDA can be a promising solution to introduce precision oncology based on complex molecular profiling data into the routine care of pediatric cancers. Notably, our program impacted clinical decision-making in over half of all cases. It is also important to mention that incorporating DDA as a personalized decision support system in pediatric patients with cancer did not delay proper oncological treatment.

Standardization is a universal need in precision oncology [53]. Notably, MTBs generally have low concordance rates, 40%–63%, from the same input data [44, 45, 54]. The use of DDA can overcome this discordance and accelerate and standardize variant interpretation and decision support. Additionally, DDA also enables eventual reinterpretation or reanalysis of the results by different criteria. We show that narrowing the definition of benefit to identifying actionable targets with clinical evidence underestimates the potential clinical utility of comprehensive genomic analysis. The presented data reveal that genomic alterations outside of ESCAT II/III categories can also have a significant impact on therapy selection by the MTB if computed aggregated evidence supports the use of a molecularly targeted agent. This is a pioneering study showing that the majority of the detected mutations would not have been covered by small targeted molecular testing (50–100-gene panel) as part of routine clinical care, and this lack of information can impact targeted therapy selection. Moreover, even larger, 400–800-gene panels would not have captured the highest evidence-level driver mutation in around 10% of patients. Of course, the virtual analysis method has limitations. We ignored (1) alterations of potential extra genes included in panels but not part of our 995-gene WES interpretation list, and (2) panel CNVs and translocations were not included in this study, raising the possibility that panel sequencing could have identified additional alterations. On the other hand, we disregarded hotspot panel detection limitations for rare, out-of-hotspot region mutations. With the increasing pace of new scientific discoveries, improvement and time-reduction of WES analysis, and rise of DDA systems, WES/WGS is expected to further outdistance panel sequencing in performance and clinical utility. Moreover, there is reasonable urgency to utilize the most comprehensive analysis with minimal tissue requirements to avoid tissue exhaustion by directed testing [55]. The use of computational tools is a logical next step to help targeted therapy selection in a standardized way based on complex genetic information. Additional benefits include comparability of results from different institutes and identification of drug classes to be prioritized for drug development and clinical trials. Taken together, in line with other studies [20, 56, 57], our results support the need for comprehensive molecular testing in pediatric malignancies.

As with any analysis, our study also has limitations. First, data come from 100 patients from a single country, which might limit the generalizability of the results. However, the case count was not much different from the median patient number (111) of studies listed in Table 1. Second, the collection of information on actual therapy administration and outcome was not part of the study. Limitations on therapy administration might impact the number of patients benefiting from therapeutic decision support. Although outcome analysis was not intended to be a part of our study, it is noteworthy that many patients did not reach the point of targeted therapy administration. One reason for this was that many patients were heavily pretreated and presented extensive metastatic lesions; therefore, in accordance with other publications [20, 24, 34, 58], our results advocate molecular diagnostic testing early in the disease course. Another reason is the limitation in accessibility of off-label treatments. Future clinical studies evaluating the clinical efficacy of treatments recommended by the DDA in pediatric cancers are warranted.

In conclusion, we demonstrate the value of DDA-based decision support as a practical input for MTBs to enable informed therapeutic stratification and improved diagnosis for children with cancer. The study revealed actionable findings, and a high percentage of top-ranked therapies was approved by the MTB. Importantly, a high AEL score correlated with MTB decisions to use matching targeted therapy, supporting the utility of the system. Data reanalysis revealed similarities with and differences from ESCAT tiering and shortcomings of panel sequencing coverage. In conclusion, we demonstrate the value of DDA-based computational interpretation of complex molecular profiling results in a structured and standardized way to integrate personalized therapeutic decisions into pediatric cancer care.

## Supplementary Information

Below is the link to the electronic supplementary material.

Supplementary file1 Table 1 Anonymized patient-related and general analysis information, driver list per case and virtual panel filtering results (XLSX 576 KB)

Supplementary file1 (MP4 20190 KB)

## Data Availability

All data generated or analysed during this study are included in this published article (and its supplementary information files) in a pseudonymized way.
